# Editorial: Unleashing the power of large data: models to improve individual health outcomes

**DOI:** 10.3389/fdgth.2025.1668543

**Published:** 2025-08-04

**Authors:** Hyewon Jeong, Sanjat Kanjilal, Sherry H. Yu, Akshay Kothakonda

**Affiliations:** ^1^ Department of Electrical Engineering & Computer Science, Computer Science and Artificial Intelligence Laboratory (CSAIL), Massachusetts Institute of Technology, Cambridge, MA, United States; ^2^ Department of Population Medicine, Harvard Pilgrim Healthcare Institute and Harvard Medical School, Boston, MA, United States; ^3^ Optima Dermatology, Macedonia, OH, United States; ^4^ Department of Otolaryngology-Head and Neck Surgery, Massachusetts Eye and Ear Infirmary and Harvard Medical School, Boston, MA, United States

**Keywords:** digital health data, machine learning, personalized medicine, big data, biomedical signal, electronic health record - (EHR), public health, predictive modeling

**Editorial on the Research Topic**
Unleashing the power of large data: models to improve individual health outcomes

The digital transformation of healthcare has unleashed unprecedented volumes of data from electronic health records (EHRs), wearables, social media, and beyond. These big data assets, coupled with advances in artificial intelligence (AI) and machine learning (ML), promise to drive precision health. However, key challenges remain in data quality, integration, and model interpretability. This Research Topic brings together interdisciplinary research that illustrates how data-driven models can improve health outcomes. They span efforts to ensure high-quality EHR-based research, novel ML applications for mental health and telehealth, data-driven clinical decision support, and techniques for making complex models interpretable and transparent.

Ensuring data availability and research framework are fundamental for turning big data into reliable health insights. EHRs represent a rich source of observational data, but their effective use involves addressing critical challenges. Honeyford et al. highlights that biases, missing data, and privacy constraints should be addressed by an iterative approach to research protocol development. They also emphasize the necessity of establishing robust platforms for ethics oversight, data quality assurance, and analytical rigor. To that end, curating and maintaining publicly available datasets empowers the research community to build upon existing work and advance the field. Wang et al. have created and shared a large dataset for vital signs, namely *PulseDB,* including over 5 million synchronized waveform segments and demographic metadata for each subject. This work has the potential to facilitate standardized evaluation of cuff-less blood pressure estimation. The study by Hong et al. promotes appropriate medication use by mining large-scale medication knowledge graphs from medical records and clinical text, and by building sequence generation models to predict medication regimens.

Sources of healthcare big data that provides clinical intuition are not only limited to in-hospital data, but extend to more non-traditional sources such as social media platforms such as X/Twitter, or clinic attendance data. Tumaliuan et al. developed a two-stage depression symptom detection model using multi-lingual data from social media (X/Twitter), demonstrating how digital traces of language and behavior can indicate mental health status. Their approach detects whether a tweet contains any sign of depression and then classifies the symptom type (e.g., sleep issues, appetite change, suicidal ideation, etc.) for English and Filipino tweets, which shows a potential for a scalable public health surveillance. Telehealth represents another non-traditional but data-rich area ripe for research. Snoswell et al. used inverse reinforcement learning to understand outpatient preferences between telehealth and in-person visits using clinic attendance data. Insights from this work can help tailor appointment options and reduce missed visits, showing how behavioral modeling can drive patient-centered care.

As ML models become more prevalent in healthcare, their interpretability and transparency remain paramount for clinical adoption. Building explanations or white-box ML techniques are crucial across all medical domains, where trust and verification of automated findings are required before they inform patient care. Yamga et al. applied unsupervised learning to multimodal COVID-19 data to identify patient phenotypes stratified by risk profile, demonstrating the potential to support targeted management early in a patient's hospital admission. In the emergency care domain, Sulaiman et al. employed rule extraction techniques with gradient boosting to predict emergency department length of stay (ED-LOS), generating human-readable rules that distill the model's decision logic for clinicians. Another study used visual interpretability techniques in deep learning for medical imaging. Liapi et al. applied transfer learning on carotid ultrasound images to classify symptomatic atherosclerotic plaques and used class activation maps (CAMs) to highlight the image regions (e.g., calcification or lipid cores) driving the model's predictions.

Across these studies, integrating diverse data—such as social media posts (Tumaliuan et al.), vital signs, and prescription logs—enhances personalized interventions and early detection. Social media analysis can identify mental health risks (Tumaliuan et al.), while phenotype clustering helps tailor treatments (Yamga et al.). Big data and machine learning can proactively address issues like emergency department crowding (Sulaiman et al.) or appointment adherence (Snoswell et al.). However, data quality (Wang et al., Hong et al.), interpretability (Yamga et al., Sulaiman et al., Liapi et al.), and equity (Honeyford et al.) are crucial to effectively translating innovations into real-world impact. Looking forward, multidisciplinary collaborations must refine models to ensure clinical relevance and generalizability. Improving data interoperability through standards, conducting prospective implementation studies, and training clinicians in AI interpretation are essential. Policymakers should encourage innovation while maintaining accountability by setting benchmarks for data quality, transparency, and fairness. These efforts will harness large-scale data responsibly, driving precision health and preventive care, ultimately improving outcomes for individuals and communities.

